# Quantifying the Impact of Mounted Load Carrying on Equids: A Review

**DOI:** 10.3390/ani11051333

**Published:** 2021-05-07

**Authors:** Syed S. U. H. Bukhari, Alan G. McElligott, Rebecca S. V. Parkes

**Affiliations:** 1 Department of Veterinary Clinical Sciences, Jockey Club College of Veterinary Medicine and Life Sciences, City University of Hong Kong, Hong Kong, China; habukhari2-c@my.cityu.edu.hk; 2 Department of Infectious Diseases and Public Health, Jockey Club College of Veterinary Medicine and Life Sciences, City University of Hong Kong, Hong Kong, China; alan.mcelligott@cityu.edu.hk; 3 Centre for Companion Animal Health, Jockey Club College of Veterinary Medicine and Life Sciences, City University of Hong Kong, Hong Kong, China

**Keywords:** blood chemistry, donkey, equid welfare, horse, limb biomechanics, loading, performance

## Abstract

**Simple Summary:**

The overloading of equids has become an important issue among veterinarians, trainers, riders, and welfare advocates. Increased weight carrying may have negative effects on biomechanical, physiological, biochemical, and behavioral parameters of equids during exercise, including causing gait asymmetry or lameness. It is important to determine how to carefully quantify the load-carrying capacity of both ridden horses and working equids. There are many options to assess the effect of loading on an animal’s body, but these have been inconsistently applied, making it difficult to reach a consensus, even for horses. This review summarises current knowledge of the load-carrying ability for horses and donkeys and the different parameters used to determine the effect of loading on these equids. Further research is needed to develop evidence-based guidelines for maximum loading in equids. Quantified loading limits or indicators of overloading could be used by stakeholders working with sports and pleasure horses and working equids to limit overloading and to improve the welfare of these animals.

**Abstract:**

There are approximately 112 million working equids in developing countries, many of which are associated with brick kilns. Brick kilns and overloading are associated with welfare problems in working equids. Understanding equids’ abilities and influencing factors are important for both effective performance and welfare. Traditionally, measurement of the amount of ‘bone’ was used, and more recently, gait symmetry has been identified as a potential marker for loading capacity. Assessment of stride parameters and gait kinematics provides insights into adaptations to loading and may help determine cut-off loads. Physiological factors such as the ability to regain normal heart rates shortly after work is an important tool for equine fitness assessment and a more accurate measure of load-carrying capacity than absolute heart rate. Oxidative stress, plasma lactate, and serum creatine kinase activity are reliable biochemical indicators of loading ability. For monitoring stress, salivary cortisol is superior to serum cortisol level for assessment of hypothalamus-pituitary-adrenal axis and is related to eye temperatures, but this has yet to be interpreted in terms of load-carrying ability in equids. Further research is needed to standardize the evidence-based load-carrying capacity of working horses and donkeys.

## 1. Introduction

**Figure 1:**
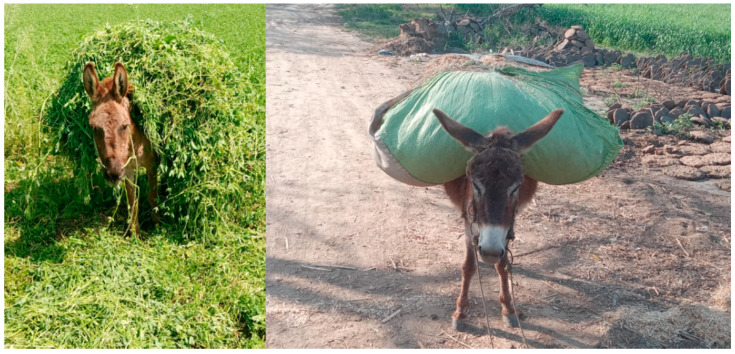
Working donkeys involved in the transportation of agricultural load (Lucerne and wheat straw) in central Punjab-Pakistan. Photo: Syed Saad Ul Hassan Bukhari.

Horses (*Equus caballus*) and donkeys (*Equus asinus*) are ridden and used globally for pleasure, sport, and transport. Equids have always had an important role, especially in developing countries, in load-carrying [1,2], transport, draught, and agricultural production [3,4] (Figure 1). There are an estimated 112 million working equids in developing countries [5]. For example, countries that have large working equid populations include China, Mexico, Ethiopia, Pakistan, and India, with 15.1, 12.9, 9, 5.5, and 1.5 million working equids, respectively. The importance of working equids is well known, but no research has quantified the amount that they are worth to the economies of these countries [5]. Although their power has been superseded by machinery in many developed countries, they remain as relevant as technology in some regions of the world because animal power is cheaper and easier to maintain compared to motorized modern power [6].

**Figure 2:**
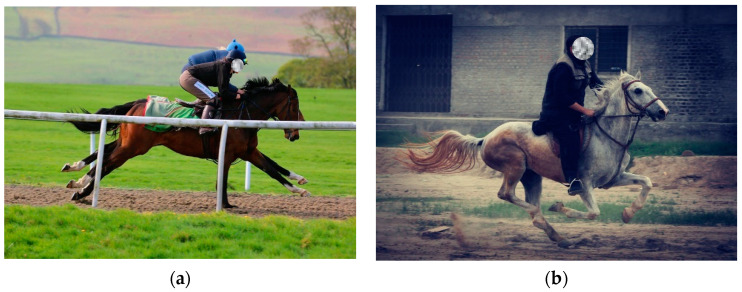
Horses with their riders: (**a**) A horse and a rider with BWR 11.96% in the UK. Photo: Mick Atkins; (**b**) A horse and a rider with BWR 24.21% in Faisalabad, Pakistan. Photo: Sabir Hussain.

Overworking and overloading have been reported as the most important issue in working horses and donkeys [7,8,9]. Overloading is defined as the weight with which gait rhythm is disrupted [10], leading to lameness and alteration of behavior [11]. Equines, especially donkeys, may be subject to overloading. This is a welfare concern [12] that requires improvement [1,2]. For ridden equids used for both work and pleasure, increased human bodyweight [11] is a potential welfare problem because people are getting heavier [13]. Obesity rates are increasing in human populations [14,15,16,17,18] as the prevalence of overweight adults increases, from 1981 to 1996, among men and women from 48% to 57% and 30% to 35%, respectively [15]. The increase in a rider’s weight (Figure 2) affects biomechanical, physiological, biochemical, and behavioral parameters of equids [10,11,19,20,21,22]. For working equids, carrying heavy loads is associated with increased income for their owners, leading to overloading by economic necessity [23].

The effect of a rider’s bodyweight (BW) on the health, performance, and welfare of horses is frequently debated in studies of horse-rider relationships [13]. Increased rider weight has a negative effect on biomechanical, physiological, biochemical, and behavioral parameters of horses during exercise [11,21,22,24]. The weight a horse can carry is important, and it depends upon a number of physical traits, including size, age, body condition score, body conformation, duration of work, third metacarpal bone circumference, type of work, and the intensity of the work to be performed [24]. Overall this subject is poorly studied [24], and most research has been undertaken on Icelandic horses, which are traditionally subjected to a high rider:horse bodyweight ratios as compared to larger Warmblood horses and are also exposed to higher exercise intensities than ordinary riding horses [25]. One factor that should be considered is that the impacts of mounted and harnessed loads are different, as the former is more energy demanding for a loaded trotting horse [26]. This explains why a horse moves slower carrying than pulling a given weight at a specific gait [27]. However, research results are conflicting [11,25], and many different measures have been used to assess weight carrying capacity [10,19,20,24,28], making direct comparisons between studies challenging.

Donkeys differ from horses in a number of biomechanical, physiological, biochemical, and behavioral respects, and they are often undervalued in the equine world [29]. For example, in comparison to horses, donkeys have closer limbs and more upright hooves and are more suited for movement over difficult terrain instead of moving at speed. Donkeys have straight backs, low withers, and slow and smooth paces, which make them ideal for load carrying [29], although donkeys have also been used for playing polo [30]. Donkeys have a steeper dorsal hoof wall angle [31], the frog is placed more caudally, and they have 25% higher mean integument depth at the level of the third phalanx as compared to horses [29]. Sick donkeys may not appear outwardly sick; instead, they may be stoic, depressed, dull, and show reduced interest towards their environment and companions [29]. Their ears are less mobile and show less response to noises. Most of the time, they lower their head below the withers in case of ill health [29]. Lameness in donkeys is difficult to assess because it is often subtle and, when laminitic, they do not show the classic ‘laminitcs stance’ that is seen in horses [29]. In addition to this, working donkeys are often reluctant to trot on demand, making assessing lameness harder [32]. These differences should be considered when comparing donkeys with horses. Not much research has been carried out on donkeys, and there is no accurate and science-based permissible load carrying limit for them. However, there is evidence that overloading in these animals is common and leads to significant welfare issues such as lameness and back pain [33]. To optimize equine welfare, people using equine power should understand their limitations. The efficient use of equines depends on understanding their capabilities for work, which can influence their optimum field performance. This review discusses the biomechanical, physiological, biochemical, and behavioral effects of loading in load-carrying equids.

### The Role of Equids in Developing Countries

In developing countries, rural people rely on working equids in agriculture, construction, and transportation for both goods and people [5]. Equids play an important role in livelihoods in various countries [34], including Kenya [35], Nepal [12], Mexico, Ethiopia, India, Pakistan, and China [5]. In particular, a large number of working equids are owned by poor people engaged with brick production in India, being an important source of income for these landless people [36]. Brick kilns mostly rely on equids, but the sex and species of the equids vary between countries and brick kilns. In India, Pakistan, Afghanistan, and Nepal, around 380,000, 115,000, 6900, and 2200 animals work in the brick kiln industry, respectively [12].

There are a number of causes of poor working equine welfare, for example, high workload, improper shelter, food, water, handling (whipping and poor driving), harmful practices (nostril slitting), lack of supporting infrastructure (good farriers, saddlers, and healthcare), marginalisation, harsh environmental conditions, lack of inclusion in legal systems and program enforcement [5]. Overloading of equids is one of the many issues that may lead to reduced welfare, which is a global concern [37]. Brick kiln work appears to be associated with greater welfare problems in working equids compared to other sectors, although the severity, range, and patterns of welfare and health issues vary between countries and brick kilns within a country [12]. The welfare of working equids should be improved through collective actions of the equid-owning communities along with organizations supporting them [34,38,39].

**Figure 3:**
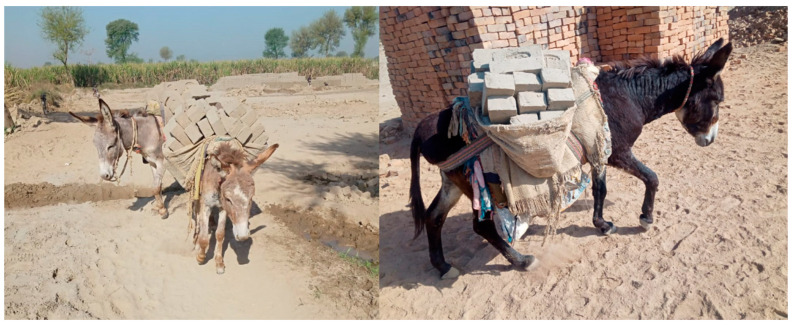
Brick kiln donkeys involved in the transportation of bricks in southern Punjab, Pakistan. Photo: Syed Saad Ul Hassan Bukhari.

Overloading is a problem in all sectors employing working equids, and it is common for equids to collapse under an overly heavy load of bricks [12]. Overloading leads to sprains, back sores, wounds, fractures, and other irreparable injuries in working equids [12], decreasing their work output and reducing their contribution to rural livelihoods [8]. Most of the work of horses and donkeys consists of the transportation of dry and wet bricks from a brick kiln to their destination (Figure 3). In India, Pakistan, Afghanistan, and Nepal, donkeys carry 100–120 kg, 120–135 kg, 125–150 kg, and 60–80 kg (on average) of bricks on their back during a single trip, respectively [12]. For comparison, the mean live weight of Pakistani donkeys is 115 kg (range, 67–153 kg) [40] and the weight of indigenous Indian donkeys ranges between 110–142 kg [28]. Every day, equids carry tons of weight, which likely exceeds their natural weight carrying capacity. This is a welfare concern, and this situation becomes further undesirable due to poor management and husbandry practices leading to musculoskeletal issues [5]. Lack of knowledge and understanding about brick kilns and working equids is mainly due to insufficient research and lack of scientific data; existing data is confusing and mainly extracted from related research that cannot be directly applied to working equines [12].

## 2. Physiological Effects of Loading

Physiological indicators such as heart rate [21,24,28,41,42], rectal temperature [21,28,41], respiratory rate [21,28], hematocrit [21,28], cost of energy [43,44], and muscle factors [24,45] have been studied in relation to the loading capabilities of horses and donkeys. However, the number of studies is not sufficient for detailed knowledge of the effect of load on physiological parameters of equids. Compared to horses, donkeys have different resting body temperature (36.5–37.7 °C), heart rate (31–53 beats/min.), and respiration rate (13–31 breaths per min) ranges. Moreover, normal respiration rates change with changes in workload and environmental temperature. When compared to horses, donkeys’ red blood cells and packed cell volume are normally lower, whereas mean corpuscular volume is higher in donkeys [29]. Most of the studies (on the impact of loading) are done on horses, and data from them may not be applicable to working donkeys.

Generally, heart rate increases with higher loads [41,42]. This change is often linear [21], and an increase of 54–59 beats/minutes occurs with a load of 25–30% BW of the horse [24]. Smaller increases in the mounted weight from 12–23% of the horse’s bodyweight do not result in a significant alteration in heart rate during a five-minute and twenty-second exercise test [25]. In donkeys walking at speeds of 0.42 to 0.55 ms^−1^ (for a maximum duration of 6 h), pulse rate increases by 18.87, 21.44, and 20.38 (min^−1^) with increasing mounted load from 40%, 50%, and 66% BW, respectively. However, the pulse rate again decreased with a load of 66% BW [28]. The ability to regain normal heart rate (resting heart rate) shortly after exercise is an important tool for equine fitness assessment [46] and may be a more accurate measure of load-carrying capacity than absolute heart rate. The type of load carried does not appear to affect heart rate, with no difference between horses carrying a rider and an equivalent weight in lead, which may reflect a minimal influence of the rider on the horse’s physiological response while exercising [42], although how the rider sits can influence performance at high speeds [47].

In the process of converting stored energy into mechanical energy during exercise, horses are inefficient and lose 80% of their stored energy as heat. Moreover, they have a high metabolic capacity and relatively small surface area to dissipate heat [48]. Horses show no change in rectal temperature while increasing mounted load from 10% BW to 20% BW [41], but an increase in rectal temperature occurs as weight is increased from 20% to 35% BW [21]. A rise of 0.3 °C, 0.2 °C, and 0.1 °C superficial temperature of the back of the trunk, front of the trunk, and neck of horses occur with an increase in load to 20% BW as compared to 10% [41], but it has been shown that cow’s superficial temperature decreases in acute stress conditions [49]. Donkeys show an increase in rectal temperature of 1.63 °C, 1.03 °C, and 1.77 °C with mounted loads of 40%, 50%, and 66% bodyweight, working at speeds of 0.42 to 0.55 ms^−1^ (for a maximum duration of 6 h), respectively [28].

In Icelandic horses under mounted load, respiration rate increases linearly with increasing load. An increase of 39 to 86 breaths/minute has been noted with an increase in mounted load from 20% to 35% BW [21]. Breathing frequency during exercise is limited by stride frequency at a gallop; therefore, breathing is important for post-exercise catch-up in Thoroughbred racehorses [50]. In donkeys, an increase in respiration rate by 28.86, 23.62, and 25.37 breaths/minute has been seen from pre-work to post-work with a mounted load of 40%, 50%, and 66% BW, respectively. However, post-work change among the three loading groups was not significant [28]. This relationship between respiration rate and mounted load suggests that the maximum permissible load in donkeys is not 50% BW, and assessing the permissible load via the post-work responses may be a more valid approach to estimating load-carrying capacity in some instances.

It is well known that exercise, irrespective of its type, changes the blood parameters of horses [51,52] and donkeys [28]. In horses, mounted weight does not have any effect on hematocrit (Hct) percentage apart from being reduced (Hct = 45%) after exercise with a 20% BW load as compared with a 35% BW load. However, this change usually reverts back to normal within 30 min (Hct = 36) after exercise in Icelandic horses [21]. Donkeys differ [29], because their hematocrit (%) increased by 3.18, 2.95, and 6.82, hemoglobin (g/dL) increased by 1.59, 1.58, and 1.72, red blood cells (million/mm^3^) increased by 0.83, 0.8, and 0.95 while white blood cells (×1000/mm^3^) increased by 1.99, 2.02, and 2.91 after work (for a maximum duration of 6 h) with 40%, 50%, and 66% BW ratio, respectively [28]. In horses, following exercise, adrenaline causes the release of erythrocytes from the spleen into general circulation [53]. It is likely that a similar mechanism occurs in donkeys, but differences between horses and donkeys in the context of loading have yet to be investigated.

In horses, loading increases the metabolic cost of transport. For example, the addition of 85 kg load, which represented 19% of bodyweight, increased metabolic rate by 18% [44]. However, some studies in humans were unable to detect an increase in metabolic rate with vertical loading from 5% to 10% bodyweight [54]. Interestingly, the energy cost of carrying a load per unit weight of load decreases with increasing weight. In horses, the energy cost is 5.8, 3.8, and 3.7 joules per kilogram per minute, while in donkeys, the energy cost is 6.5, 4.4, and 3.0 joules per kilogram load per minute, for the weights of 13, 20, and 27 kg/100 kg live weight, respectively. There is no difference between horses and donkeys [43]. Moreover, the cost of energy per unit load is the same whether weight is of additional load or the body alone [55] and an increase in speed causes increased energy consumption as compared to carrying a heavy load [56].

## 3. Biomechanical Assessment

Measurement of the amount of ‘bone’ (i.e., the circumference of the third metacarpal bone or ‘cannon bone’) is a traditional method to determine the weight carrying ability of a horse [57,58,59]. Several studies have used cannon bone measurements for the evaluation of skeletal biomechanical relationships, for example, the relation of bone strength to bone structure, stress, tension, and elasticity [57,58,59]. Horses with a larger cannon bone circumference and greater loin width have a greater load-carrying ability [24]. Heavy horses without a proportional increase in third metacarpal bone circumference had a higher incidence of biomechanical collapse [60], but for donkeys, this has yet to be assessed. Further studies for depth of loin muscles, the total area of loin, the usefulness of subjective evaluation of muscle soreness/tightness, and serum creatinine kinase (an enzyme representing the extent of skeletal muscle damage amid load carrying) activity are required to explain the biomechanical relationship of equids with mounted weight.

Horses adjust their gait from walk to trot or gallop at a point that minimizes energy consumption, thus managing the cost of locomotion [61]. They have a preferred speed for each particular gait type to minimize musculoskeletal stresses and enhance the metabolic economy. Loading increases musculoskeletal stresses and metabolic rate leading to a decreased preferred speed in trotting horses [44]. However, this has not been quantified in donkeys.

### 3.1. Gait Stability and Symmetry

Gait stability is defined as the sum of gait regularity (similarity on contralateral steps and similarity of consecutive strides) and gait symmetry. At a normal trot, a whole stride consists of a pair of similar dorsoventral motions, where one peak of correlation must be at each time corresponding to each half stride. Therefore, the first two peaks provide two coefficients that quantify symmetry of gait and gait regularity [19,20,62]. Gait symmetry is related to lameness, with animals demonstrating gait asymmetry above a certain threshold, sometimes adjusted for breed or use, considered to be lame [63,64,65,66]. Gait symmetry and load-induced lameness have also been used as markers for loading capacity [10,19,20], and it has been suggested that carrying an inappropriately high weight may induce lameness in horses [11]. Trot symmetry decreases with increased weight carried in Japanese horses [19]. This effect is not seen during walking [20]. Gait symmetry is reduced, and lameness has been observed in horses of mixed breeds, with rider weights of up to 27.5% horse BW [11], but not in another using rider weights of up to 23% horse BW [25].

While trotting, gait stability decreases significantly when carrying 120 kg compared to 70 kg in Taishuh ponies [19], and in Yonaguni ponies (Japanese Landrace horses) carrying 70 kg compared with no load [10]. However, Taishuh ponies are generally small in size, with bodyweights between 216 kg and 265 kg [19]. Yonaguni pony bodyweight varies from 192 kg to 227 kg [10]. Again, during walking, no difference in gait stability was observed in Japanese native horses when carrying loads [20], and this may be due to low speed during a walk. Gait stability as a measure of load-carrying capacity has not been used by other authors in assessing horse and donkey gait.

### 3.2. Stride Parameters

In order to explain locomotor changes quantitatively, both angular stride variables (such as fetlock range of motion and maximum fetlock angle) and temporal stride variables (swing duration, relative stance, and stride duration) are important [67]. Assessment of stride parameters and gait kinematics provides further insight into biomechanical adaptations to loading and may help determine cut-off loads. Stance time is likely to be particularly important because stance time as a proportion of stride time gives duty factor, which is a useful proxy for peak vertical ground reaction force; therefore, limb load [68]. Stance time is the period of time during which the foot is in contact with the ground surface [69], stride time is the time elapsed between the first contact of two consecutive footsteps of the same foot [70], duty factor is the fraction of stride for which the foot remains on the ground [71], and ground reaction force is the force exerted by the ground on the body during contact [72]. Fetlock flexion is also directly proportional to limb load [73] and, therefore, may also be useful in assessing adaptations to load carrying. Limb load is important in this context because the peak loading capacity of the limbs is thought to limit speed in horses [74]. By increasing the load carried by the animal, the load carried by each individual limb is also increased, and if the load applied is sufficient and not limited by other factors, peak limb loading limits may be reached.

For horses moving on a treadmill, stride duration becomes longer (as stride frequency becomes 1.6% slower), carrying a load of 19% BW [75]. An increase in stance duration has been seen in Dutch Warmblood horses under-mounted rider or dead weight compared to an unloaded situation [42]. In Icelandic horses, an increase in the bodyweight ratio (from 20% to 35%) causes a decrease (from 2.63 m to 2.58 m) in stride length and increase in duty factor (from 40.8% to 43.2%) proportionally to the same extent in all four limbs [22]. Foot contact time increases by 7.7% in horses carrying loads (19% BWR) on a level surface, but no difference has been found for an incline under the same conditions [75].

An increase in mounted load predictably leads to an increase in ground reaction force at the trot. This is greater in the front legs than the hind legs, which may be due to changes in rider position at the trot, thus changing the center of mass [76]. During short-term exercise, the rider’s bodyweight does not affect the gait biomechanics of Warmblood horses [25]. An increase in step length has been observed in Arabian horses at trot [45,75]. In Icelandic horses, a decrease in stride length (from 2.63 to 2.58 m) and an increase in stride frequency (from 2.09 to 2.12 strides/s) have been seen with additional weight from 20% BWR to 35% BW [22]. However, the differences between tölt (running walk, a gait used by Icelandic horses) and trot should be kept in mind when interpreting the differences in their comparative biomechanical studies. The trot is a two-time gait with the bodyweight of the horse usually supported on a diagonal pair of limbs at any one time, while at the tölt, the horse often has tripedal or even quadrupedal support [77], therefore reducing the maximum load on each individual limb. A tölting horse has a briefer stance duration, higher stride rate, lower stride impulses, shorter stance duration in forelimbs, higher peak vertical force in forelimbs, longer stance duration in hindlimbs, lower peak vertical force in hindlimbs, and comparatively higher head-neck position that is the cause of no weight shift towards the hindlimbs. There is also a higher forefoot flight along with lower protraction. However, variation in stride to stride timings are greater in tölt as compared to trot, but all limbs during trot typically present a spring-like mechanism [77]. Different authors have employed different measures under a variety of conditions for assessing gait changes in response to load-carrying [10,11,19,20,22,25,75]. This lack of consistency means that, at present, it is difficult to formulate accurate calculations for weight carrying ability in horses or donkeys based on biomechanical changes.

## 4. Changes in Blood Biochemistry

A large number of biochemical and enzymatic factors such as blood lactate, creatinine kinase, malondialdehyde, nitrates, nitrites, glutathione, retinol, tocopherol, cholecalciferol, lactate dehydrogenase, and cortisol level have been studied in relation to the loading abilities of equids [21,24,25,28,42,78]. However, the number of studies is not adequate for optimum coverage of the effect of load on biochemical parameters of equids.

Plasma lactate concentration is a reliable parameter for the measurement of the effect of load [79]. Plasma lactate level increases (from 3.6 to 7.9 mmol/L) exponentially with the increase in the horse:rider bodyweight ratio from 20% to 35%. Plasma lactate concentration returns to its normal level (0.9 mmol/L) at 24 to 48 h post-exercise, which is much slower compared to heart rate recovery. Aerobic metabolism of lactate is more efficient with a 20% bodyweight ratio compared to a 35% bodyweight ratio, indicating a more efficient recovery [21]. Horses mainly work aerobically until the bodyweight ratio becomes 22.7%. However, individual differences exist (17–27.5%), which may be linked to body condition score rather than body size. The size of the animal is not the decisive factor of how much an animal can carry [21]; overall body condition score is more important than the bodyweight ratio for performance [80]. Plasma lactate level immediately after exercise and ten minutes post-exercise was lower in horses carrying 15%, 20%, and 25% bodyweight ratios compared to a 30% bodyweight ratio [24]. There was no difference found between mounted and lead loaded horses; however, in that particular investigation, horses carried only 12.6% and 16.3% of their bodyweight [42]. In loaded donkeys, plasma lactate concentrations were higher in all 40% (1.03 mmol/L), 50% (1.20 mmol/L), and 66% (1.14 mmol/L) bodyweight groups as compared to pre-exercise concentration (0.92–0.99 mmol/L), but a greater increase was seen only in 50% bodyweight group during post-exercise tests. Plasma lactate did not increase in 40% and 66% (as in 50% BW) bodyweight groups, but this may be because of the low speed of work [28].

The rise in serum creatine kinase enzyme activity is a useful indicator of post-exercise muscle soreness and muscle damage in humans [81], as well as skeletal muscle damage in horses [82]. Serum creatine kinase activity was higher immediately post-exercise and 24 to 48 h post-exercise when animals carried a weight of 30% bodyweight ratio, but no change in enzyme activity was seen when horses carried a load of 15% and 20% bodyweight ratio [24]. Interestingly, activities of plasma creatine kinase, twofold above resting level (400 µ/L), corresponded with a negligible amount of muscle damage [83], adding other factors which may be more important for the determination of weight-bearing ability than serum creatine kinase activity.

There is evidence of oxidative stress in horses while doing endurance and intense exercise [84]. The importance of the relationship between oxidative tissue damage and physical activity has been studied in humans and animals. As metabolism increases due to physical activity, this leads to increased production of free radicals by electron transport systems and cellular antioxidant systems giving their response to these free radicals. Malondialdehyde is a naturally occurring organic compound and is an oxidative stress marker in biological systems [78,85,86,87]. The levels of malondialdehyde increase (from 0.78 to 1.22 nmol mL^−1^) after work with heavy loads in equids and provide evidence of disturbance of cellular oxidative homeostasis [78]. The damage at the cellular level is controlled and prohibited by various bio-mechanisms working through many chemicals, including tocopherol and glutathione [88], and the blood level of tocopherol decreases (from 4.8 to 3.6 µg mL^−1^) and glutathione increases (from 10.6 to 20.8 mg dL^−1^) after work with mounted load (equivalent to 30% of their bodyweight) in healthy horses, their levels were measure using spectrophotometric methods [78]. Muscles contain a large number of receptors for cholecalciferol (Vitamin D), which helps in calcium and phosphate metabolism. Numerous studies have demonstrated the relationship of cholecalciferol to physical performance [89], and it has been demonstrated that blood level of vitamin D decreases (from 0.1 to 0.03 µg mL^−1^) with mounted load associated work in equids, its level was measured using high-performance liquid chromatography (HPLC) [78].

## 5. Behavioral Measures and Indicators of Stress

The use of behavioral indicators for the assessment of load-carrying capacity in equines is in its infancy. An equine ethogram has been used to assess pain-associated ridden behaviors in horses [90], which may be useful when used by trained assessors [91]. More recently, a grimace scale for pain has been developed for use in donkeys [92], although this has not yet been used in the field. No ethogram for use in working donkeys has yet been developed, although overall behavior is included in several existing welfare assessment frameworks [37]. The validity of ethograms for use in working donkeys may be questionable, as some working equids exhibit apathy and reduced responsiveness, likely due to sickness or chronic poor welfare conditions [93]. The use of the ridden equine ethogram has demonstrated that heavier riders can induce behaviors associated with musculoskeletal pain (e.g., reluctance to move forward, tail lashing, and constant movement of head with ears back) and temporary lameness in horses [11]. Various breeds of horses were used, including Warmblood, Thoroughbred, Cob type, Irish Sport Horse, and Lusitano. If we include breed factors into weight-bearing capabilities, then this could be a limiting factor for this particular study as different breeds of horses may have different loin width and amount of ‘bone’ which have been shown to be important factors for weight carrying ability [24]. However, in another recent study, no changes in behavioral signs were detected with the increase in mounted weight from 12% to 23% BW of the horse during low-intensity exercise (5 min and 20 s exercise test) [25]. Moreover, heart rate variability (vagal regulatory activity estimating high beat to beat change) was lower with heavier (20% BW) riders as compared to lighter (10% BW) riders [41], but heart rate variability is yet to be investigated in donkeys.

Biochemically, stress in horses can be characterized by an increased amount of exhaled nitrogen monoxide (NO) [94]. NO is a biological messenger and synthesized from amino acid (L-arginine) and, in a very short period of time, oxidizes to Nitrite (NO_2_) and Nitrate (NO_3_), which make it difficult to measure NO levels precisely. The concentration of NO also increases in certain pathological conditions and acts as a free radical [78,95,96,97]. Likewise, the concentration of nitrite and nitrate also increases (NO_2_ 0.78 to 0.89 ppm; NO_3_ 2.01 to 4.16 ppm), but a concentration of retinol (vitamin A) decreases (0.50 to 0.18 µg mL^−1^) in response to work associated with load carriage up to 30% of the bodyweight in horses. These findings provide sufficient evidence that intensive work increases oxygen consumption, ultimately disturbing cellular antioxidant and pro-oxidant balance [78]. The measurement of salivary cortisol is much better than serum cortisol level for assessment of hypothalamus-pituitary-adrenal activity as it eliminates the need to care for between-subject differences in cortisol binding globulin or within-subject changes [98]. However, it is understood that cortisol is strongly linked with psychological and emotional states [99,100,101,102,103]. Moreover, a relationship has been found between eye temperature, salivary and plasma cortisol levels suggesting that eye temperature may also be associated with activation of hypothalamus-pituitary-adrenal activity in animals [104,105], but regarding load carrying, this should be interpreted by keeping in mind gait symmetry and behavioral data [25]. Cortisol is not a good measure of work and load-related stress because it may also be significantly affected by diet [106], genetic factors [107], environment, and characteristics associated with individuals [108]. Generally, it is considered that cortisol level increases with workload and stress, but in recent research on horses, it has been demonstrated that during short term exercise (a 5 min and 20 s exercise test), an increase in rider’s weight by 15% to 25% did not result in an increase in cortisol level [25].

## 6. Weight Limitations

At this stage of scientific research, in this particular field of equine science, answering the questions related to mounted load limitations for horses and especially donkeys is difficult. This is due to the use of different methods and procedures used in different studies under different conditions [10,11,19,20,21,22,24,25,28,75]. Thus, a conclusive comparison for weight carrying ability is currently nearly impossible for equids.

It has been demonstrated that increased rider weight can have a negative effect on biomechanical, physiological, biochemical, and behavioral parameters of horses during work [11,21,22,24]. However, one more recent study showed that increasing mounted weight from 12% to 23% BW of the horse did not result in significant changes in heart rate, gait symmetry, behavior, and cortisol level during low-intensity work (5 min and 20 s exercise test) [25]. These conflicting recent results could be resolved by doing more detailed research on single horses with different riders with similar weight and exercise intensity to study the effect of riders beyond their weight.

The maximum permissible load carriage for native Japanese horses (bodyweight 339.9 ± 37.5 kg) has been suggested as less than 100 kg, a number established after studying gait parameters, which is about 29% of an adult horse bodyweight [19,20]. On Yonagunai ponies (Japanese Landrace horses), it was hypothesized that a gradual increase in load on trotting ponies will lead to the point of biomechanical compromise, i.e., change in gait symmetry and regularity. They established that maximum permissible load carriage for trotting Japanese land race ponies is less than 70 kg, which is 33% of its BW [10], and for Taishuh ponies (Japanese small breed horse) permissible load carriage is less than 100 kg (about 43% of its BW) trotting at a speed of 3.00 ms^−1^ [19]. However, to improve welfare, it is vital to find out loading capacity for individual breeds of horses that vary in average bodyweight [10]. Individual variations in the proportion of muscle fiber can affect metabolic responses [109], and genetics could have an important role in the weight carrying capacity of horses [21].

For donkeys, little research has been done measuring load-associated changes in physiological and biochemical parameters. It was concluded that Indian indigenous donkeys could safely carry loads up to 50% bodyweight [28]. Interestingly, lactate levels did not increase for loads of 40% and 66% compared to results for loads of 50% bodyweight [28]. Most importantly, biomechanical and behavioral measures were not considered in this study, and the results shown are based on physiological and biochemical parameters, while biomechanical and behavioral responses are yet to be investigated. Donkeys working on United Kingdom beaches are limited to carry not more than 51 kg [110], representing approximately 28% of their BW. However, this recommendation is based on experience rather than scientific evidence. Additionally, these donkeys are doing short rides at a slow speed [111] on a fairly level surface with a good body condition score. Working donkeys in other contexts are often working on uneven or steep terrain [12,29]. In donkeys, load density does not affect the cost of transport [43]; however, incline does affect metabolic responses and work rate [112].

## 7. Conclusions

Overloading of equids is one of the many issues that may seriously compromise their welfare. Over the last three decades, much work has been done to understand the effect of mounted load on the performance of horses, but its effect on donkeys is less studied. We know that different Japanese horse breeds can carry 29–43% of their bodyweight, but no similar research has been carried out for other horse breeds or donkeys. Loading affects a large number of biomechanical, physiological, biochemical, and behavioral features of equines, and further research is required to advance our current understanding of these factors. Quantified loading limits or indicators of overloading could then be used by NGOs, policymakers, and other stakeholders working with vulnerable communities and working equids to limit overloading and to improve the welfare of these animals.

## Data Availability

Not applicable.
